# Resolution of an insidious and migratory *Mycobacterium tuberculosis*-associated secondary organizing pneumonia: a case report and literature review

**DOI:** 10.1186/s12879-023-08334-5

**Published:** 2023-06-01

**Authors:** Li-Li Huang, Chun Wang, Ying Liu, Xiao-Yan Gu, Wei-Xiao Wang, Wei Chen, Chun-Mei Hu

**Affiliations:** 1 Department of Tuberculosis, the Second Hospital of Nanjing, Affiliated Hospital to Nanjing University of Chinese Medicine, 1-1 Zhongfu Road, Gulou District, Nanjing, 210003 China; 2 Clinical Research Center, the Second Hospital of Nanjing, Affiliated Hospital to Nanjing University of Chinese Medicine, 1-1 Zhongfu Road, Gulou District, Nanjing, 210003 China; 3 The Clinical Infectious Disease Center of Nanjing, Nanjing, 210003 China

**Keywords:** Secondary organizing pneumonia, *Mycobacterium tuberculosis*, Misdiagnosis, Cryptogenic organizing pneumonia, tNGS

## Abstract

**Background:**

Organizing pneumonia (OP) is a rare interstitial lung disease. Secondary organizing pneumonia (SOP) caused by *Mycobacterium tuberculosis* (MTB) is extremely rare. Migratory MTB-associated SOP is more deceptive and dangerous. When insidious tuberculosis (TB) is not recognized, SOP would be misdiagnosed as cryptogenic organizing pneumonia (COP). Use of steroid hormone alone leads to the progression of TB foci or even death. Clues of distinguishing atypical TB at the background of OP is urgently needed.

**Case presentation:**

A 56-year-old female patient was hospitalized into the local hospital because of cough and expectoration for more than half a month. Her medical history and family history showed no relation to TB or other lung diseases. Community-acquired pneumonia was diagnosed and anti-infection therapy was initialized but invalid. The patient suffered from continuous weigh loss. More puzzling, the lesions were migratory based on the chest computed tomography (CT) images. The patient was then transferred to our hospital. The immunological indexes of infection in blood and pathogenic tests in sputum and the bronchoalveolar lavage fluid were negative. The percutaneous lung puncture biopsy and pathological observation confirmed OP, but without granulomatous lesions. Additionally, pathogen detection of the punctured lung tissues by metagenomics next generation sequencing test (mNGS) were all negative. COP was highly suspected. Fortunately, the targeted next-generation sequencing (tNGS) detected MTB in the punctured lung tissues and MTB-associated SOP was definitely diagnosed. The combined therapy of anti-TB and prednisone was administrated. After treatment for 10 days, the partial lesions were significantly resorbed and the patient was discharged. In the follow-up of half a year, the patient was healthy.

**Conclusions:**

It is difficult to distinguish SOP from COP in clinical practice. Diagnosis of COP must be very cautious. Transient small nodules and cavities in the early lung image are a clue to consider TB, even though all pathogen tests are negative. tNGS is also a powerful tool to detect pathogen, ensuring prompt diagnosis of TB-related SOP. For clinicians in TB high burden countries, we encourage them to keep TB in mind before making a final diagnosis of COP.

**Supplementary Information:**

The online version contains supplementary material available at 10.1186/s12879-023-08334-5.

## Background

Organizing pneumonia (OP) is an interstitial lung disease, histologically characterized by inflammatory debris that fill the alveoli, alveolar ducts, and terminal bronchioles. The organized polypoid granulation tissue is also known as the Masson bodies[1–3]. From the perspective of etiology, OP could be categorized into two types: cryptogenic organizing pneumonia (COP) and secondary organizing pneumonia (SOP). The former does not have specific cause for the disease, but the latter has. It’s worthy of note that OP is a nonspecific inflammatory response to lung damage and both COP and SOP share similar clinical, radiographic and pathological findings and treatment response [4]. The etiologic diagnosis in patients with OP is very important because the primary underlying disease must be managed. If SOP is misdiagnosed as COP, only corticosteroid therapy would be initiated, which might lead to rapid dissemination of the original infection lesion. To date, many causes of SOP have been identified, such as infection, drug toxicity, eosinophilic pneumonia and other interstitial lung diseases [2, 5]. *Mycobacterium tuberculosis* (MTB) is rarely reported to be an infectious cause of OP. In this study, we described a case of SOP with insidious and migratory pulmonary TB lesions.

## Case presentation

**Fig. 1:**
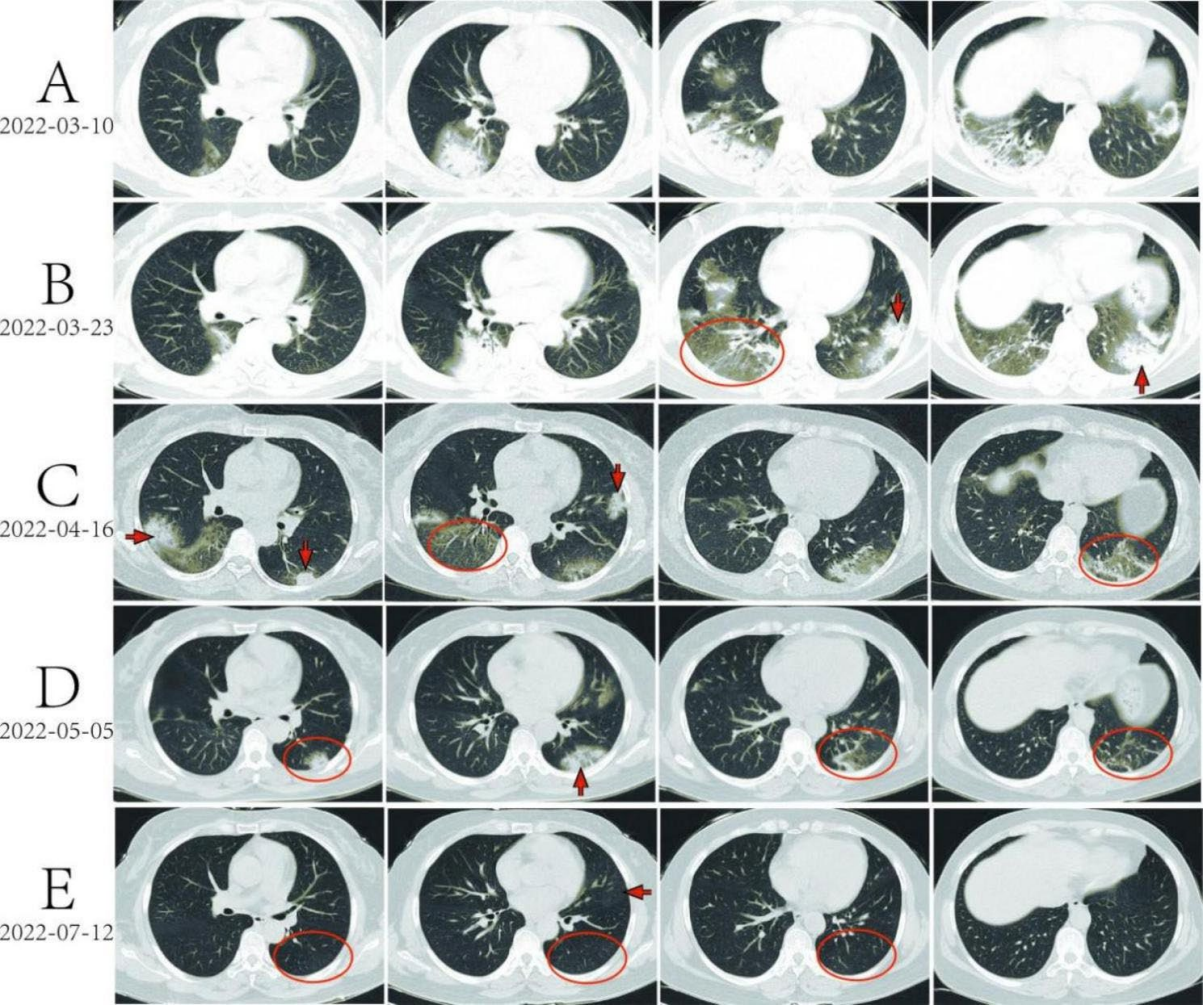
Chest CT scan at different time-points. **(A)** Chest CT before treatment. Multiple high-density patchy shadows were observed in the middle and lower lung fields of both lungs, with air bronchogram, partial bronchiectasis and unclear lesion boundary. Small alveolar cavity was observed in the lesion of superior segment of right lower lobe. Calcification foci was absent. **(B)** Chest CT After 2 weeks of anti-infective treatment. The lesion was progressed, particularly in the basal segment of the left lower lobe as the focal point (arrow). The lesion on the right lung was absorbed and the patchy ground glass density shadow remained in the right lower lobe. **(C)** Chest CT in our hospital. Multiple patchy and rope-like high-density shadows were observed near the pleura in both lungs, with bronchial vapor phase visible, partial bronchiectasis and unclear lesion boundaries inside. New lesions can be observed in both lower lobe and left upper lung (arrow), while the lesion in the basal segment of the left lower lobe was absorbed (circle). **(D)** Chest CT after 10 days’ treatment combined of anti-TB and corticosteroid therapy. Multiple patchy and rope-like high-density shadows were observed in both lungs, with air bronchogram, partial bronchiectasis and unclear lesion boundaries. The lesion was slightly enlarged (arrow) and slightly absorbed in left lower lobe (circle). **(E)** Chest CT at the follow-up of two month. Multiple patchy and rope-like high-density shadows were observed in both lungs, but significantly improved compared with Fig. 1D(circle). A small localized ground-glass density shadow was observed in the lingual segment with unclear boundary(arrow). There was no specific abnormal density foci in the other lobe

A 56-year-old female patient, weighing 70 kg, was admitted to a local hospital due to “cough and expectoration for more than half a month” on Mar 11, 2022. Two weeks ago, the patient had shown symptoms of paroxysmal cough, moderate phlegm, accompanied with night sweats and fatigue after catching a cold, while no obvious purulent sputum, bloody sputum, chills, fever, muscular soreness, chest tightness or chest pain. The patient still coughed severely after taking the cold medicine and lost 1.5 kg weight. On Mar 10, 2022, the chest computed tomography (CT) showed multiple flake-like lesions in both lungs, which were considered to be infectious lesions (Fig. 1A). The personal history (including environmental exposure history) and family history showed no relation to the lung disease, and there were no obvious positive signs in physical examination. Biochemical and coagulation functions were normal. The white blood cell count was 10.06✕10^9^/L, the neutrophil ratio was 74%, and the C-reactive protein was 65.48 mg/L, all of which were significantly increased. These examinations led to a preliminary diagnosis of community-acquired pneumonia at the local hospital and the patient was treated with Amiloride and Cefodizime IV as anti-infection therapy, which was then changed to Piperacillin/Tazobactam and Levofloxacin IV after one week. At the beginning, the patient’s cough was relieved, but one week later, she developed a recurrent cough with paroxysmal exacerbation, mild chest tightness after exercise and intermittent night sweats. 5 kg weight was lost. The lesions in the lower right lung were partially resorbed. However, new lesions appeared in both posterior and lateral basal segment of left lower lung in the chest CT (Fig. 1B).

The patient was transferred to our hospital on Apr 14, 2022, and her vital signs were as below: body temperature, 36.2 °C; pulse, 78/min; respiratory rate, 18/min; blood pressure, 124/77 mmHg. On the next day, she completed routine blood, biochemical and coagulation index tests, and immunological indexes of infection in blood (Table S1), as well as pathogenic tests in sputum (Table S2). All the results were within the normal range or negative. To further seek the pathogenic diagnosis, a bronchoscopy was performed on Apr 18, 2022. Lavage was performed in the outer basal segment of the left lower lobe of the lung, and the lavage fluid was sent for detection of pathogenic tests for bacteria, fungi and virus by a pathogenic metagenomics next generation sequencing test (mNGS). All the results were negative (Table S3). No tumor cells were found in the pathological tests of the lavage fluid. Chest CT suggested that the lesions were resorbed in the right lower lung and posterior segment of left lower lung. However, the lesions were aggravated in the apical segment of left lower lung and lateral segment of right middle lobe (Fig. 1C).

**Fig. 2:**
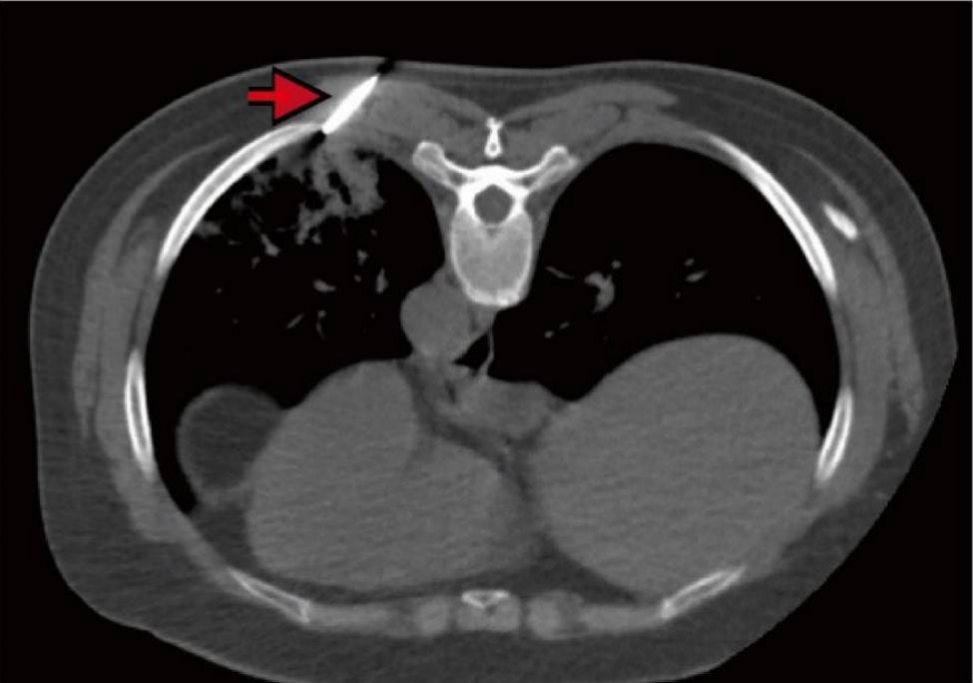
Transverse section of CT-guided percutaneous lung biopsy. The position of the puncture needle is indicated by the arrow

**Fig. 3:**
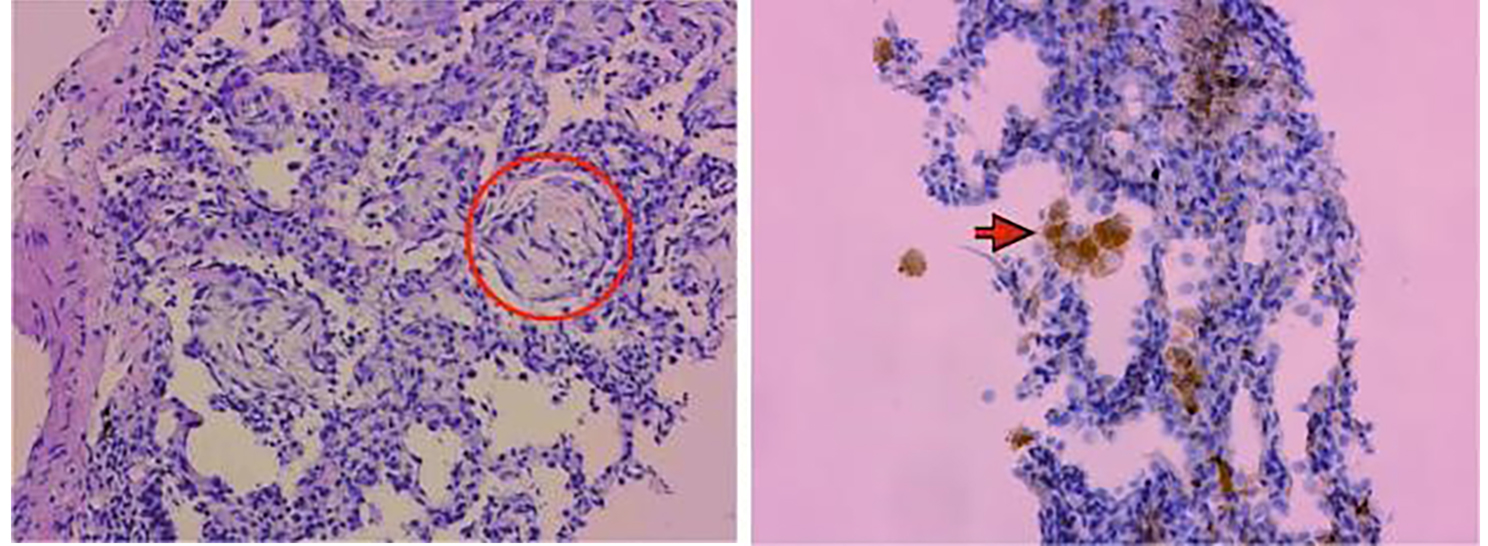
Masson body and CD68 staining. Biopsy pathological section revealed Masson body indicated by a red circle (left). CD68 staining showed that CD68 positive cells were only observed in the alveolar lumen, as indicated by the red arrow (right)

On Apr 20, 2022, a percutaneous lung puncture biopsy was performed on new lesions in the left lower lung (Fig. 2), and the pathology report suggested that striated lung tissue showed chronic inflammation with widened alveolar septa, and focal areas showed the structure of Masson bodies, which was typical pathological changes of OP (Fig. 3). The immunohistochemical results of cytomegalovirus were negative. Both of periodic acid-schiff (PAS) and acid-fast staining were negative. Further refinement of CD68 staining showed that the CD68-positive epithelioid cells were all located in the alveolar lumen (Fig. 3), which suggested that the tissue had not yet formed granulomatous lesions. Combined with the dynamic imaging findings, the pulmonary lesions were more likely considered as infectious lesions. To clarify the infection-related factors, we determined the punctured lung tissues by mNGS, and all the results were negative (Table S4).

At that stage, sputum and bronchoalveolar lavage fluid had been tested negative for etiology, and mNGS of lung tissue had not detected a clear causative agent. Additionally, the patient denied the history of aspiration, connective tissue diseases (CTDs), organ transplantation, and other diseases. Taken together, COP was highly suspected.

Fortunately, the punctured lung tissues were simultaneously sent for targeted next-generation sequencing (tNGS) of MTB, a novel pathogenic microbial detection method based on ultra-multiplex Polymerase Chain Reaction (PCR) and second-generation sequencing technology, which enables simultaneously identification of MTB and analysis of drug resistance genes. The results showed that MTB was detected with an abundance of 6.38% and a relative concentration of 8.99✕10^2^ with a medium confidence strength.

Accordingly, the patient was finally diagnosed as pulmonary TB and SOP, and was given isoniazid, rifampin, ethambutol, and pyrazinamide for anti-TB treatment. Meanwhile, prednisone, 40 mg per day, was administrated. On May 5, 2022, the chest CT before discharge showed the lesions in the left lower lung slightly progressed while the remaining lesions were significantly resorbed (Fig. 1D). During anti-TB treatment, the sputum cultures for MTB were negative.

On Jun 4, 2022, at the follow-up of one month, the patient reported that the cough and sputum symptoms were significantly relieved. The prednisone dose was reduced to 30 mg per day. On Jul 12, 2022, at the follow-up of two months, the latest chest CT showed significant improvement in absorption compared with the previous CT (Fig. 1E). Only localized ground-glass density shadow remained in the lingual segment of the upper lobe of the left lung, and no new lesions were found. On Nov 4, 2022, the patient mentioned that her general health status was satisfactory in the telephone follow-up.

## Discussion

### SOP caused by MTB is rare

OP is a pathological concept of the mechanization of inflammatory exudates in the alveolar lumen, alveolar ducts, and respiratory fine bronchi with fibroblast and myofibroblast proliferation. Fibrous foci within the alveoli are called Masson vesicles and may be accompanied by lymphocyte, plasma cell, and monocyte infiltration [6]. Actually, OP is the end product of the lung tissue repair process and is associated with a variety of diseases such as CTDs, various drugs, infections, malignancies, radiation exposure, post-transplantation, and other interstitial pneumonia. Infection-related pathogens of SOP are mostly viruses (e.g., H1N1 influenza A virus), bacteria (e.g., Aureus), cryptococcus, and aspergillus [7, 8]. SOP caused by MTB infection is very rarely reported (Table S5) [9–12]. One possibility is SOP itself is a rare disease, with an incidence rate 1.1-7 cases/100,000 [7, 13]. The other possibility is due to the small amount of MTB in the lesion, which is hard to detect by routine TB-related pathogen tests.

### Our MTB-associated SOP case lacks typical pathological and radiological characteristics of TB

The occurrence mechanism of OP associated with TB infection is unclear. It is speculated that the immune response to TB might occur in certain regions of the body, where alveolar epithelial cells are damaged, T lymphocytes and neutrophils are triggered, aggregated, and activated to secrete inflammatory mediators and cytokines, and then fibroblasts are activated, leading to granulomas formation [14]. In this case, biopsy pathology performed within the emerging lesion showed chronic inflammation with widened alveolar septa and Masson bodies structures in the focal area, while typical pathological changes of TB such as exudation, proliferation, and necrosis were not evident. Additionally, there were no typical imaging manifestations of TB, such as the tree-bud sign. This might be due to the latent or early phase of TB infection that activated the immune response, leading to mechanistic changes. Alternatively, percutaneous pulmonary puncture biopsy had its limitations that TB granulomatous changes were not present in the biopsied tissue by accident.

Different from COP and other SOPs, infection-related SOP usually has fixed foci, with less variability and less frequency of wandering, which mostly present as focal mechanized pneumonia (FOP) [7, 9–11, 14]. In contrast, the chest CT in our SOP case showed multiple patchy consolidation in both lungs, with wandering and variable lesions. The SOP lesions in our case initially appeared in the medial segment and posterior segment of the right lower lobe, then migrated to the lateral and posterior segment of the left lung. Three weeks later, the lesions migrated to the right middle lobe, and then appeared in the anteromedial segment of the left lower lobe two weeks later.

**Fig. 4:**
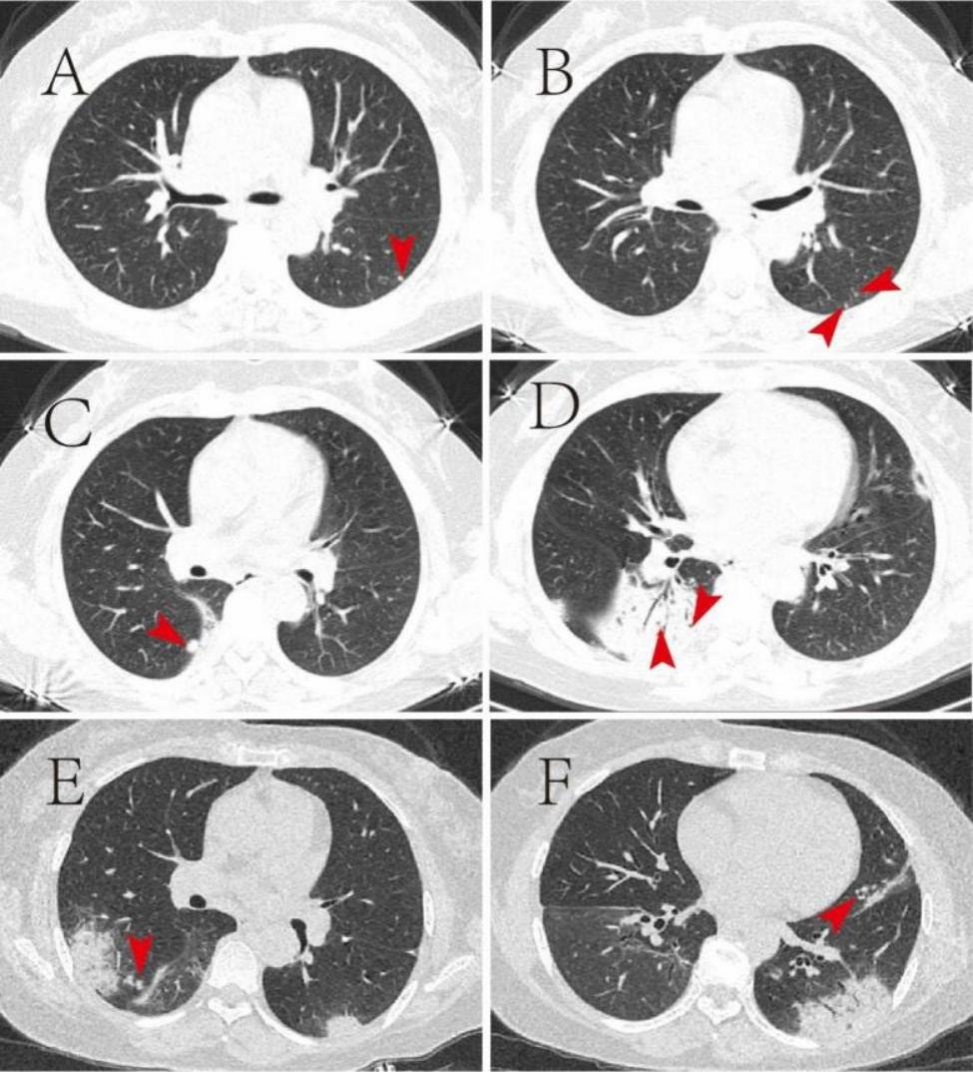
Radiological characteristics of atypical pulmonary TB. Transient small nodules **(A, B, C)**, small cavities in consolidation **(D)**, and central lobular nodules **(E, F)** are observed in chest CT taken on 16 April, 2022

When we analyzed the patient’s imaging data retrospectively, small nodules and central nodules in both lower lung lobules were seen in the periphery of both lungs in the early stage, which suggested the presence of granulomatous nodules or nodules of airway-origin. Multiple small cavities were seen inside the solid shadow of the right lower lung, which might be related to liquefaction, necrosis, and expulsion of lung tissue caused by MTB infection (Fig. 4). These provided clues for the diagnosis of pulmonary TB, suggesting that the wandering solid shadow in the lung may be a rare atypical imaging change of pulmonary TB.

### tNGS is highly-sensitive to clarify MTB-associated SOP

The gold standard for the diagnosis of pulmonary TB is acid-fast staining and culture of MTB [15]. At present, nucleic acid detection of MTB, such as GeneXpert MTB/RIF[16], mNGS and tNGS [17], has been gradually applied in clinical practice due to its high sensitivity and short duration. Because it is difficult to break the cell wall of MTB, mNGS detection is prone to be false negative. While tNGS detection is specialized for nucleic acid extraction based on the characteristics of mycobacteria, thus the sensitivity is higher. Therefore, when diagnosis and etiology are not apparent, tNGS could help to clarify MTB-associated SOP and initiate anti-TB treatment promptly.

### Combined anti-TB therapy and corticosteroid therapy

Corticosteroid treatment is not regularly recommended for patients with MTB-associated SOP. Usually, anti-TB regimen is enough. Use of corticosteroid depends on the therapeutic response of anti-TB. However, for patients with severe TB, addition of corticosteroid to anti-TB regimen resulted in significant resorption of pulmonary lesions and better prognosis [10, 11, 14, 18, 19]. In this case, anti-TB and corticosteroid treatment were initialized simultaneously based on the following considerations. Firstly, the patient had a rapid onset and progression of lesions. Secondly, the patient’s symptoms gradually developed, and chest tightness occurred. At last, anti-TB treatment has a long course and the symptom relief is relatively slow.

The lesions of the patient in our study developed during the early course of anti-TB combined with corticosteroid therapy. There are three possible explanations. At first, mechanized pneumonia and primary pulmonary TB are poorly controlled or treatment time is insufficient, with lesions still in continuous progression. Secondly, paradoxical response reaction occurred during anti-TB therapy. Thirdly, because of low immune system of the patient and continuous dissemination of TB infection lesions, the addition of anti-TB may result in immune reconstitution inflammatory syndrome-like reactions [14]. As for our case, the combination of anti-TB and short-term corticosteroid hormones was satisfactory.

## Conclusions

SOP itself is a rare disease, and MTB-related SOP reports are extremely rare. Although the imaging and pathological features of TB have different manifestations in different stages of disease, some clues are very helpful to identify TB in this case. Small nodules and central nodules in both lower lung lobules in the periphery of both lungs in the early stage, suggests the presence of granulomatous nodules or nodules of airway-origin. Multiple small cavities inside the solid shadow of the right lower lung, might be related to liquefaction, necrosis, and expulsion of lung tissue caused by MTB infection. In addition, tNGS is a powerful tool to detect pathogen, ensuring the early diagnosis of TB-related SOP. For clinicians in TB high burden countries and regions, we encourage them to keep MTB in their mind to make a final diagnosis of COP. Otherwise, the use of corticosteroid therapy may lead to the progression of TB foci or even death.

## Electronic supplementary material

Below is the link to the electronic supplementary material.

Supplementary Material 1

## Data Availability

Data relating to this study are contained and presented in this document. Other materials are available from the corresponding authors on reasonable request.

## Abbreviations

OP: organizing pneumonia
SOP: secondary organizing pneumonia
MTB: *Mycobacterium tuberculosis*
TB: tuberculosis
COP: cryptogenic organizing pneumonia
tNGS: targeted next-generation sequencing
CT: computed tomography
PAS: periodic acid-schiff
mNGS: metagenomics next generation sequencing test
CTDs: connective tissue diseases
PCR: polymerase Chain Reaction
FOP: focal mechanized pneumonia
CRP: C-reactive protein
ESR: erythrocyte sedimentation rate
