# Supplementary material for: Resolution of an insidious and migratory Mycobacterium tuberculosis-associated secondary organizing pneumonia: a case report and literature review
Source: BMC Infect Dis. 2023 Jun 1;23:372. doi: 10.1186/s12879-023-08334-5 (PMC10236784; doi:10.1186/s12879-023-08334-5)
Supplement: Supplementary file 1 — Supplementary Material 1 [file 12879_2023_8334_MOESM1_ESM.pdf]

Table S1. General information and blood related tests.

Age/Gender	56/Female
Symptom	Cough, and phlegm
Past history	Negative
Blood routine	
Leukocyte number	8.11×10^{9}/L
Neutrophils	60.6%
The inflammatory index	
Procalcitonin	Normal
C-reactive protein (CRP)	16.01 mg/L
Erythrocyte sedimentation rate (ESR)	45 mm/hr
Bacteria-related tests	
Respiratory pathogen IgM	Negative
T cell test for tuberculosis infection	Negative
Tuberculosis antibody	Negative
Fungal related detection	
Detection of β-D-glucan and galactomannan	Negative
Detection of cryptococcus capsular antigen	Negative
Allergen IgE detection	Negative
Autoimmune antibody related detection	
Antinuclear antibodies	Negative
Vasculitis-related antibodies	Negative

Table S2. Pathogen detection of sputum.

**Bacteriology-related test**	
Culture of common bacteria	Negative
**Fungi-related detection**	
Fungal culture	Negative
**MTB-related detection**	
Smear for acid-fast bacillus	Negative
MTB DNA	Negative
GeneXpert MTB/RIF	Negative

Table S3. Pathogen detection of alveolar lavage fluid.

**Bacteriology-related test**	
Culture of common bacteria	Negative
**Fungi-related detection**	
Fungal culture	Negative
Detection of β-D-glucan and galactomannan	Negative
**MTB-related detection**	
Smear for acid-fast bacillus	Negative
MTB RNA	Negative
MTB DNA	Negative
GeneXpert MTB/RIF	Negative
**Pathological smear for tumor cells**	Negative

Table S4. Detection of pathogens in the lung punctured tissues by mNGS.

Bacteria	Negative
Mycobacterium	Negative
Mycoplasma/chlamydia	Negative
Fungi	Negative
DNA virus	Negative
Parasite	Negative
Resistance genes	Negative
Virulence genes	Negative

Table S5. Cases of MTB-associated SOP.

	Age/Gender	Underlying diseases	Symptoms	Chest CT	Treatment	Prognosis	Reference
**Case 1**	27/Female	HIV-infected, previous pulmonary TB	Cough, fever and right-sided chest discomfort	N/A	Anti-TB chemotherapy	Died	[9]
**Case 2**	75/Male	Hypertension, atrial fibrillatio, history of colon polyp surgery	Cough, phlegm, dyspnea with moderate fatigue, fever and joint myalgia	Ill-defined alveolar infiltration, lobular mass and a solid nodule in RLL, and pleural effusion	Anti-TB chemotherapy and corticosteroids	Symptom improved	[10]
**Case 3**	78/Female	None	Cough	Multifocal consolidation in the RUL, RLL, and LLL, with small nodules	Anti-TB chemotherapy	Recovered	[11]
**Case 4**	78/Female	None	Cough with yellowish sputum production and chest pain	Ground-glass opacities and multifocal consolidation with air bronchogram in the posterior segment of the RUL	Anti-TB chemotherapy	Recovered	[11]
**Case 5**	81/Male	Hypertension	Anorexia, fever, cough, and phlegm	Lobar consolidation in both upper lung fields, focal consolidation in RML and RLL, and bilateral pleural effusion.	Anti-TB chemotherapy	Recovered	[12]
**Case 6**	72/Male	Neurofibromatosis, gastrointestinal stromal tumor, previous pulmonary TB	Cough, phlegm	Consolidation in the LLL	Anti-TB chemotherapy	Recovered	[12]
**Case 7**	54/Female	Diabetes, hyperlipidemia	Fever	Identification of a speculated mass in RUL	Anti-TB chemotherapy	Recovered	[12]

**Case 8**	57/Male	None	Generalized weakness and dyspnea	Multiple micronodules distributed randomly in both lungs, accompanied by cavitary nodules, irregular linear opacity, and patchy consolidation in both apices.	Anti-TB chemotherapy	Recovered	[12]
**Case 9**	78/Male	None	Fainting	Suspected pulmonary edema with diffuse patchy consolidations and GGO with a crazy paving pattern in both lungs.	Anti-TB chemotherapy	Recovered	[12]
**Case 10**	70/Male	None	Asymptomatic	A solitary pulmonary nodule	Anti-TB chemotherapy	Recovered	[12]

Note: N/A, not available; LLL, left lower lobe; RUL, right upper lobe; RLL, right lower lobe; RML, right middle lobe; GGO, ground glass opacity.
